# Mental well-being during stages of COVID-19 lockdown among pregnant women and new mothers

**DOI:** 10.1186/s12884-021-04374-4

**Published:** 2022-02-01

**Authors:** Gritt Overbeck, Ida Scheel Rasmussen, Volkert Siersma, Jakob Kragstrup, Ruth Kirk Ertmann, Philip Wilson

**Affiliations:** 1 The Research Unit for General Practice and Section of General Practice, Department of Public Health, University of Copenhagen, Copenhagen, Denmark; 2 Centre for Rural Health, Institute of Applied Health Sciences, University of Aberdeen, Aberdeen, Scotland

**Keywords:** Anxiety, Depression, Pregnancy, Covid-19, Cross-sectional

## Abstract

**Background:**

Pregnancy and early motherhood are sensitive times where epidemic disease outbreaks can affect mental health negatively. Countries and health care systems handled the pandemic and lockdowns differently and knowledge about how the COVID-19 pandemic affected the mental well-being of pregnant women and new mothers is limited and points in different directions.

**Aim:**

To investigate symptoms of anxiety and depression in a population of pregnant women and new mothers in various stages of infection pressure and lockdown during the first 15 months of the COVID-19 pandemic in Denmark.

**Methods:**

The study population was nested an inception cohort of women recruited in their first trimester of pregnancy. Data about mental health of the woman were obtained in relation to pregnancy and child development (first trimester, 8 weeks postpartum and 5 months postpartum), and data were analysed cross-sectionally according to calendar time (periods defined by infection rate and lock-down during the COVID-19 pandemic).

**Results:**

No differences in reported levels of depressive symptoms between the six examined time periods of the pandemic were observed. Specifically, symptoms remained unchanged after the first lock-down. No major changes in anxiety symptoms were observed in relation to increased infection pressure or lockdowns, but a small increase was observed during the second lockdown in women 8 weeks postpartum.

**Conclusion:**

No clear change in mood among pregnant women was seen between during the stages of COVID-19 pandemic in Denmark.

## Introduction

Pregnancy and early motherhood are sensitive times where epidemic disease outbreaks can affect mental health negatively [1, 2]. A feeling of vulnerability may be caused by the risk of infection posed to the child and the mother, but also by an increased need for a well-functioning health care system in pregnancy, childbirth and the early life of the child. In the first months of the COVID-19 pandemic, it was uncertain to what extent the infection would affect the unborn child and the pregnant woman’s physical condition. At the same time the health care system underwent profound changes due to COVID-19 infections, introducing rules about social distancing, and the needs for preventive procedures in health care and in society.

Knowledge about how the COVID-19 pandemic affected pregnant women and new mothers is still fragmented. A systematic review of mainly Chinese studies found slightly elevated anxiety levels [3]. Similarly, we found minor changes in symptoms of depression and anxiety among Danish pregnant women in the early stage of the pandemic [4], whereas a cross-national study including data from Norway, Switzerland, Netherlands and UK found high levels of depressive symptoms and generalized anxiety in pregnant and breastfeeding women during the COVID-19 outbreak [5]. The observed differences between studies could be due to national differences, different stages of the pandemic at the time of observation or other differences between populations. For the adaptation of a health care system to a changing epidemic within a country it may, therefore, be important to analyse the effects of various stages of infection pressure and lock down within the population of pregnant women and young mothers.

To knowledge the effects of the various stages of the pandemic have not been investigated.

We aimed to investigate symptoms of anxiety and depression in a population of pregnant women and new mothers in various stages of infection pressure and lock down during the first 15 months of the COVID-19 pandemic in Denmark. It is, to our knowledge, the first study into anxiety and depression differences between the various stages of a pandemic in pregnant women and new mothers.

## Methods

### Setting

In December 2019, news media reported a viral outbreak in Wuhan, China. In early 2020 the World Health Organisation (WHO) declared COVID-19 a global pandemic with 110,000 confirmed cases of virus infection in 110 countries. Subsequently governments all over world responded with lockdowns and other preventive measures.

**Fig. 1:**
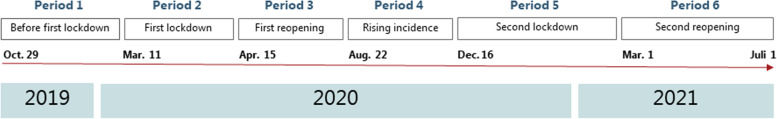
Six stages in the COVID-19 pandemic and lockdown in Denmark

In Denmark the epidemic got little publicity before March 2020. The government announced a first lockdown on the 11th of March. Schools and day-care centres were closed, and employees in the public sector and most of the private sector were sent home from work. Danish authorities encouraged social distancing from the start and set up rules for numbers meeting and travelling. Pregnant women were advised to follow the general recommendations, which in the spring 2020 and winter 2020–2021 involved social distancing. Pregnant women were also advised to follow recommendations for high risk patients which included talking to their employer about avoiding risk of infections at work. The National Board of Health recommended that pregnant women employed in the health and social sector were sent home in their third trimester [6]. From July 2021 vaccination was recommended for all pregnant women in their second and third trimester [7]. Pregnancy health consultations were considered as high priority health care and women were encouraged to take part in normal preventive care. Partners were still allowed to be present during the birth and while the mother and baby were in the hospital. As testing and personal proection equipment (e.g. face masks) was introduced in summer 2020 women and their partners were also subject to these restrictions at the hospitals. A gradual re-opening began April 15th 2020, starting with day-care, schools and the private labour market. Infection rates declined during summer, but from the star of August 2020 hospital admissions for Covid-19 patients started to increase. Due to high admission rates a second lock-down was ordered on December 22nd. On March 1st 2021 a gradual reopening was started because of falling infection rates and increasing vaccination of the population. Based on these events, the time between October 2019 to June 2021 may be divided into 6 periods (1: Before first lockdown, 2: First lockdown, 3: First reopening, 4: Rising incidence, 5: Second lockdown, 6: Second reopening). Each period represents specific risks and difficulties for pregnant women and young mothers (Fig. 1).

### Design

Our study population was embedded in an inception cohort (women recruited in the beginning of pregnancy). Data about mental health of the woman was obtained in relation to pregnancy and child development (first trimester, 8 weeks postpartum and 5 months postpartum), and data were analysed cross-sectionally according to calendar time (periods defined by infection rate and lock-down during the COVID-19 epidemic). This design made it possible to observe changes in symptoms of anxiety and depression in pregnancy and the early motherhood as a function of events in the COVID-19 pandemic.

### Participants

Pregnant women were recruited consecutively from October 2019 until June 2021 by their general practitioner (GP) at the first antenatal consultation *(between 6 and 10 weeks gestation). In total 70 GP-clinics from two of five Danish regions recruited pregnant women from urban, sub-urban and rural areas.* No exclusion criteria were used, but knowledge of Danish language was necessary. Participants gave informed consent to take part in a cluster randomized trial designed to evaluate an online psycho-educational program designed for use by all pregnant women The randomized trial was the sampling frame for our cohort of women, but the trial design and content of the intervention [8] is unlikely to have important consequences for the present study of COVID-19.

### Data

After informed consent, three electronic questionnaires were sent to the women in the first trimester, 8 weeks postpartum and 3 months postpartum. A secure electronic mail system (e-Boks) was used to approach participants about the survey, and questionnaires were completed and returned into the study database (REDCap) [9]. Two reminders were sent in relation to each of the three questionnaires if they had not been received within 2 weeks. Answers received before June 1st. 2021 were included in the study. We also received a copy of the pregnancy health record from the GP by means of REDCap.

The level of depression and anxiety was assessed by the Hospital Anxiety and Depression Scale (HADS). HADS is a standardized questionnaire which has been validated in a number of studies [10, 11]. It was developed for patients with somatic conditions, but it is often used as a self-rating scale to screen for anxiety and depression symptoms in the general population and across a range of patient groups [10]. The instrument prioritises mental symptoms, rather than physical symptoms that can be confused with pregnancy-related symptoms or physical illness. The self-completed HADS contains 14 items in two subscales: anxiety (HADS-A) and depression (HADS-D), each with seven items. Each item is rated on a four-point scale from 0 to 3 (3 indicating maximum symptom severity), and the scores are summed [9]. The scale has been translated into Danish and has proved to have high internal consistency in a large sample of Danish patients with cardiac disease [12]. Cronbach’s alpha for the anxiety sub-scale was found to be .68 to .93 (mean .83) [10] and for the depression sub-scale .67 to.90 (mean .82) [10] Changes in HADS score have been considered clinically important if they are > 1.5 points [13]. HADS was administered with the baseline questionnaire in the first trimester, again at 8 weeks postpartum and a third time at 5 months postpartum.

We obtained information about the age of the women (≤25, 26–30, 31–35, > 35 years) and cohabitation status (single/ living with partner) from the pregnancy health record provided by the GP. The electronic patient questionnaire obtained in the first trimester contained information about occupational status (employed/ student/ unemployed/ sick leave/other) and whether other children were living at home (no/yes).

### Statistical analysis

Cronbach’s alpha was calculated to assess internal consistency of the two HADS scales (anxiety and depression) at each of the three assessments. Also for each of the three assessments, the mean of the two HADS scales (anxiety and depression) were calculated in each of the six defined periods and plotted with corresponding 95% confidence intervals on a time line. In each of the graphs a moving average based on penalized B-splines was superimposed; the transparency proportional to the uncertainty. The means of the different periods were compared to the mean in period 5 (second lockdown) in linear regression analyses adjusting for age, number of children in the household and employment status. Period 5 five was chosen because at that time responses from women in all three phases of motherhood were available and because it a priori could be expected to be the period of maximum stress (second lockdown).

## Results

Table 1 shows the characteristics of the women in the study group at the three points in early motherhood where they answered the questionnaire. All questionnaires answered before July 1, 2021 were included for analysis. In average the response rate was 71.6%. Among 804 women, who received a questionnaire in first trimester, 669 (83%) answered, 222 women out of 343 (65%), who received it at 8 weeks postpartum and 170 of 254 (67%) returned the questionnaire at 5 months postpartum.

**Table 1:** Characteristics of women in the study group at the three points in early motherhood

	First trimester	Eight weeks postpartum	Five months postpartum
	*(n = 669)*	*(n = 222)*	*(n = 170)*
	n (%)	n (%)	n (%)
Period			
1	240 (35.9)		
2	60 (9.0)		
3	172 (25.7)	10 (4.5)	
4	101 (15.1)	108 (48.7)	31 (18.2)
5	71 (10.6)	56 (25.2)	85 (50.0)
6	25 (3.7)	48 (21.6)	54 (31.8)
Age			
≤ 25 years	40 (6.0)	8 (3.6)	5 (2.9)
26–30 years	208 (31.1)	72 (32.4)	52 (30.6)
31–35 years	259 (38.7)	95 (42.8)	71 (41.8)
> 35 years	162 (24.2)	47 (21.2)	42 (24.7)
Number of children in the household	(1 missing)	(6 missing)	(6 missing)
No children	273 (40.9)	106 (49.1)	74 (45.1)
One child	305 (45.7)	88 (40.7)	73 (44.5)
Two or more children	90 (13.5)	22 (10.2)	17 (10.4)
Employment status	(2 missing)	(6 missing)	(7 missing)
Employed	546 (81.9)	172 (79.6)	129 (79.1)
Under education	59 (8.9)	23 (10.7)	16 (9.8)
Unemployed or sick leave	62 (9.3)	21 (9.7)	18 (11.0)

Cronbach’s alpha for HADS-A was .80, .79 and .75, and for HADS-D .76, .76 and .75 in the first trimester, 8 weeks postpartum and 5 months postpartum respectively.

Table 2 shows the differences in HADS scores in the six time periods during 15 months of the pandemic.

**Table 2:** Differences in HADS scores (anxiety and depression) between defined periods in the pandemic at three points in motherhood (first trimester of pregnancy, 8 weeks postpartum and 5 months postpartum)

	*First trimester*			*Eight weeks postpartum*			*Five months postpartum*		
	*mean diff*^*1*^ *(95%CI)*	*p-value*	*power to detect a 1.5 point difference*	*mean diff*^*1*^ *(95%CI)*	*p-value*	*power to detect a 1.5 point difference*	*mean diff*^*1*^ *(95%CI)*	*p-value*	*power to detect a 1.5 point difference*
HADS Anxiety		0.2046^2^			0.1172^2^			0.5175^2^	
1	−0.79 (−1.66; 0.08)	0.0742	0.83						
2	0.12 (− 1.00; 1.24)	0.8334	0.61						
3	−0.78 (−1.69; 0.12)	0.0900	0.80	−0.71 (−2.92; 1.50)	0.5294	0.21			
4	−0.39 (−1.38; 0.60)	0.4411	0.72	−1.23 (− 2.30; − 0.15)	0.0251	0.68	−0.34 (− 1.57; 0.90)	0.5955	0.71
5	(ref)			(ref)			(ref)		
6	−0.14 (−1.65; 1.37)	0.8575	0.39	−0.22 (−1.55; 1.10)	0.7408	0.52	0.41 (−0.64; 1.45)	0.4457	0.86
HADS Depression		0.7888^2^			0.4883^2^			0.5667^2^	
1	−0.36 (−1.07; 0.35)	0.3224	0.95						
2	−0.46 (−1.37; 0.45)	0.3183	0.80						
3	−0.27 (−1.00; 0.47)	0.4804	0.94	−0.37 (−2.31; 1.57)	0.7103	0.31			
4	−0.15 (− 0.95; 0.67)	0.7154	0.88	− 0.67 (−1.57; 0.23)	0.1441	0.86	−0.50 (−1.59; 0.58)	0.3637	0.74
5	(ref)			(ref)			(ref)		
6	0.25 (−0.91; 1.45)	0.6852	0.55	−0.16 (−1.26; 0.94)	0.7749	0.72	0.09 (−0.82; 1.01)	0.8424	0.89

^1^adjusted for age, work status and number of children in the household

^2^*p*-value of a test for differences in mean between the six categories jointly

**Fig. 2:**
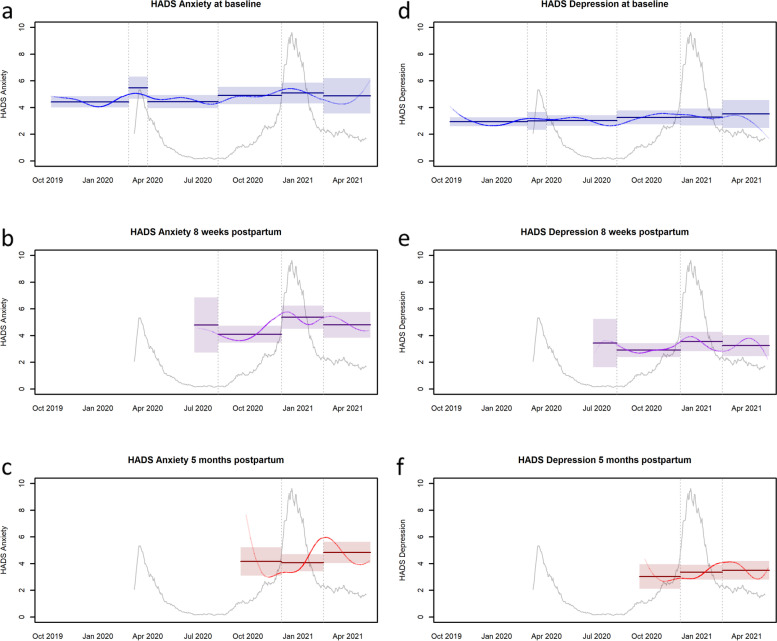
Anxiety and depression symptoms at first trimester, 8 weeks postpartum and 5 months postpartum, in relation to COVID-19-hospital admissions during observation period including stages of the pandemic

Figure 2 a-f show anxiety and depression symptoms reported by the women at first trimester, 8 weeks postpartum and 5 months postpartum, in relation to COVID-19-hospital admissions during the observation period. The vertical lines in each figure illustrates each of the six stages in the COVID-19 pandemic and lockdown.

We found no statistically significant differences in reported levels of depressive symptoms between the six examined time periods of the pandemic. Specifically, symptoms remained unchanged before and after the first lock-down.

No major changes in anxiety symptoms were observed in relation to increased infection pressure (COVID-19 hospitalizations) or lockdowns, but a statistically significant small increase of 1.23 points was observed during the second lockdown (compared to the previous 6 months) in women 8 weeks postpartum.

No statistically significant differences in the response to infection pressure and lock down were found between the two arms of the randomized controlled trial.

## Discussion

No significant differences were found in scores of anxiety and depression among the pregnant women before and after the onset of the pandemic and no major differences in scores were observed between the different stages of the pandemic among pregnant women and mothers of new-born. The only statistically significant change was an increase of 1.23 points (on a 28 point scale) in anxiety symptoms at 8 weeks postpartum during the second lockdown in the winter 2020/2021. Changes in HADS scores are considered clinically significant in individuals when they exceed 1.5 points [11], and we, therefore, consider the observed change in the population as minor.

### Strengths and limitations

To our knowledge this is the first report of changes in the mental wellbeing of a substantial population-based sample of pregnant and postnatal women during discrete phases of societal change during the COVID-19 pandemic.

The power to detect a 1.5 point difference was assessed adequate (≥80%) for many of the comparisons in Table 2. Moreover, the effects evaluated in this table are all considerable below the clinically important difference of 1.5 point, except in one place where the estimated mean difference of 1.23 is close to 1.5 point; this is also the only place where this difference is significant at a 5% level. Hence, we think it unlikely that with our data we have not found a real clinically important difference, except perhaps in the few comparisons with very low power.

We need, however, to consider a number of limitations which may lead to underestimation of the effects of the various elements in the epidemic. First of all, the various stages in the pandemic were not sharply defined. The infection rate changed continuously, and some elements of lockdown came unrelated to the major events. This may have reduced the observed differences between the stages of the pandemic in our study, but cannot explain the lack of difference in scores before and after the onset of the pandemic. Secondly, sampling in our study may have favoured well educated and employed women. The participating GPs were asked to invite all women attending their first preventive pregnancy consultation, but it has previously been shown that vulnerable women are likely to be underrepresented [12]. A similar bias is, however, likely to exist for all surveys of pregnant women and young mothers.

### Findings in context

Previous studies have shown varying effects of the epidemic on mental health and mood [14]. Some have reported high levels of depressive symptoms and generalized anxiety in pregnant and breastfeeding women during the COVID-19 outbreak [5, 15–17], while others have shown no or milder effects [4, 18]. Differences may relate to both extent of infection in societies, and handling of the pandemic [19] but also to the availability of maternal health care. Quarantine was introduced as a public measure to prevent spread of infection. People who were infected or had been in contact with infected people were asked to isolate. The psychological impact of quarantine can be wide-ranging and potentially long-lasting [20]. Denmark never faced a curfew such as that seen in some other countries and the health care system was never overwhelmed by patients with infection. The health care system, therefore, gave priority to preventive consultations and health care for pregnant women during the pandemic, and only small changes were made (e.g. increased use of internet consultations). Furthermore, since all pregnant women were considered at risk during the first 15 months of the pandemic they could stay at home without losing income. All women had paid maternity leave for at least 6 weeks before the expected date of birth and at least 24 weeks after birth. This together with the experience that health care system never broke down, because infection rate was manageable, might have served as protective factors. Our findings might therefore not be replicated in societies with a larger sickness burden from COVID 19 or a less comprehensive welfare safety net. A systematic review of risk factors during the first year of COVID-19 found that financial strain and low education were key sociodemographic factors associated with increased depression and anxiety in perinatal women [17]. “The findings in this study show that Danish pregnant women and new mothers were mentally quite robust to major societal upheaval such as the pandemic and the restrictions this entailed. This probably implies that peri- and postnatal care in Denmark functioned adequately in spite of many health care resources being allocated to COVID-19.”

## Conclusion

We could not demonstrate a clear change in mood among pregnant women between during the stages of Covid-19 pandemic in Denmark. However, since pregnancy and childbirth are vulnerable periods of life, health care providers should pay close attention to the mental health of new mothers. Potential long term consequences of the pandemic and its severe societal impact should be monitored in the coming years.

## Data Availability

The datasets used and analysed during the current study are available from the corresponding author on reasonable request.
